# Dietary Lead Exposure and Associated Health Risks in Guangzhou, China

**DOI:** 10.3390/ijerph16081417

**Published:** 2019-04-19

**Authors:** Man Wang, Boheng Liang, Weiwei Zhang, Kuncai Chen, Yuhua Zhang, Hongwei Zhou, Yanfang Cheng, Huachun Liu, Xianwu Zhong, Yingyue Li, Yufei Liu

**Affiliations:** 1 School of Public Health, Sun Yat-sen University, Guangzhou 510080, China; wangm259@mail2.sysu.edu.cn; 2 Guangzhou Center for Disease Control and Prevention, Guangzhou 510440, China; liangboheng_1999@163.com (B.L.); bin_cheng818@163.com (W.Z.); ckc@gzcdc.org.cn (K.C.); pisceszyh@126.com (Y.Z.); gzcdczhw@126.com (H.Z.); yfcheng1012@163.com (Y.C.); liuhc2019@163.com (H.L.); zhongxw.gzcdc@foxmail.com (X.Z.); gzcdclyy@163.com (Y.L.)

**Keywords:** food, lead, dietary exposure, risk assessment, margin of exposure

## Abstract

Lead exposure is associated with a wide range of adverse effects on human health. The principal exposure route in the general population is through the diet. In this study, we estimate the dietary lead intake and associated health risks among the residents of Guangzhou, China. Data on lead concentrations were derived from the food safety risk monitoring system, which included 6339 samples from 27 food categories collected in 2014–2017. Food consumption data were taken from a 2011 dietary survey of 2960 Guangzhou residents from 998 households. Dietary lead intake was estimated by age group (3–6, 7–17, 18–59, and ≥60 years), and relevant health risks were assessed using the margin of exposure (MOE) method. The mean and 95th percentiles (P95) of dietary lead intake were respectively 0.7466 and 2.4525 μg/kg body weight per day for preschool children aged 3–6 years; 0.4739 and 1.5522 μg/kg bw/day for school children aged 7–17 years; 0.3759 and 1.1832 μg/kg bw/day for adults aged 18–59 years; and 0.4031 and 1.3589 μg/kg bw/day for adults aged ≥60 years. The MOE value was less than 1 for preschool children at the mean exposure level and for all age groups at the P95 exposure level. Rice and its products, leafy vegetables, and wheat flour and its products were found to be the primary food sources of lead exposure. Our findings suggest that the health risk from dietary lead exposure is low for Guangzhou residents overall, but that young children and consumers of certain foods may be at increased risk. Continued efforts are needed to reduce the dietary lead exposure in Guangzhou.

## 1. Introduction

Lead is a heavy metal that is naturally present in the Earth’s crust. High levels of lead in the environment are primarily due to anthropogenic factors such as mining and smelting, battery manufacturing, recycling of waste batteries, burning of coal, and use of leaded petrol, leaded paints, and lead piping [1,2]. The level of lead in the environment has risen by more than 1000 times over the last three centuries due to human activity [3]. Lead has a long history in China, which is now the world’s largest producer and consumer of lead. Environmental lead pollution has become a serious problem due to the outdated production technologies in some small lead processing plants [1,4]. The Chinese government has already taken note of the severity of this situation and has adopted a series of measures to reduce lead levels. For example, the sale and use of leaded gasoline have been banned nationwide since 1 July 2000 [5], and lead standards were implemented in 2002 and 2004 for fertilizers and foods, respectively, in order to reduce lead levels in soil, foods, and packaging materials. China also signed an international agreement in 2007 which prohibits the use of lead paint in the production of toys, while also addressing other safety issues [6]. Finally, the National Development and Reform Commission proposed a series of requirements for the lead industry in 2007 [7]. Although these measures have substantially reduced environmental lead levels, lead exposure is still a substantial public health concern in China [8].

Attention has long been focused on the fact that lead is a chronic cumulative toxicant. Exposure to lead in the general population can occur through food, water, air, soil, and dust, but food is the primary source of non-occupational exposure [9,10,11,12]. Food may be polluted by lead-contaminated soil, water, and air; by metal equipment used in food production; or by food packaging materials. The use of lead-containing food additives is another source of contamination. The Global Environment Monitoring System/Food Contamination Monitoring Programme (GEMS/Food) listed lead in food as a key monitoring project and conducted systematic periodic assessments of the risks of dietary exposure to lead [13]. Lead entering the body is mostly stored in the bones and is eliminated very slowly. The biological half-lives of inorganic lead are approximately 30 days and 10−30 years in blood and bone, respectively [14]. Because of the long half-life of lead in the body, chronic harmful effects of long-term, low-dose exposure are of substantial concern. Such effects include compromised neurobehavioral development, cardiovascular disease, hypertension, impaired renal function, reproductive dysfunction, adverse pregnancy outcomes, decreased immunity, and endocrine disorders [15,16]. The most prominent and sensitive target system for lead toxicity in humans is the central nervous system, especially the developing brain [17]. A considerable body of scientific evidence shows that children are more susceptible to lead neurotoxicity than adults, and even low levels of lead exposure are associated with adverse neurobehavioral development in children [18,19,20,21,22]. The International Agency for Research on Cancer classified inorganic lead compounds as probably carcinogenic to humans (Group 2A) in 2006 [23]. The World Health Organization (WHO) has identified lead as one of the ten chemicals of major public health concern [24]. It is estimated that, in 2016, lead exposure caused 540,000 deaths and 13.9 million years of healthy life lost globally, accounting for 63.8% of the global burden of idiopathic developmental intellectual disability, 3% of the global burden of ischemic heart disease, and 3.1% of the global burden of stroke [25].

Exposure assessment is essential for quantifying risk and is a critical component of chemical risk assessment in food [26]. Results of exposure assessment can be used to judge risks to human health and to assess the effectiveness of current strategies for decreasing contaminant levels in food [27]. They can also provide information on risk characterization to support risk managers in decision-making [28]. The Food and Agriculture Organization of the United Nations/World Health Organization (FAO/WHO) have recommended three primary approaches for assessing dietary exposure to food contaminants: total diet study, duplicate diet study, and selective study of individual foods [29]. Of these three methods, the selective study of individual foods using available food consumption data and concentration data is the easiest to implement, and its results have a high degree of validity [9].

Estimates of dietary lead intake have historically been compared with health-based guidance values (e.g., the provisional tolerable daily intake (PTWI)) to assess health risk. However, in 2010, based on the latest research data, the Joint FAO/WHO Expert Committee on Food Additives (JECFA), in its 73rd meeting report, estimated that a PTWI of 25 μg/kg body weight of lead is associated with a decrease of at least three intelligence quotation (IQ) points in children and an increase in systolic blood pressure (SBP) of approximately 3 mmHg in adults. Therefore, the committee concluded that the PTWI could no longer be considered sufficient to protect health, and it was withdrawn. However, the committee was unable to establish new health-based guidelines, as there is no evidence for a threshold for the key effects of lead [30]. The margin of exposure (MOE) approach was initially recommended by the European Food Safety Authority (EFSA) to assess the risk caused by substances that are both genotoxic and carcinogenic [31]. The EFSA later recommended that the MOE method also be applied to evaluate the health risks from lead in food [32]. In 2011, Australia used the MOE approach for the first time to assess the health risks of dietary lead exposure in their 23rd total diet study [33]. In China, in 2012, Li et al. adopted the same method to evaluate the health risk of dietary lead exposure in the Chinese population [34].

Guangzhou, the capital of Guangdong province, is a large and densely populated coastal city. Because of the city’s proximity to the sea, local residents consume a relatively large amount of seafood, which usually contains high levels of heavy metals [35]. In 2008, the detection rate of lead in food in Guangzhou was 76.68%, with 8.29% exceeding standard rates [36]. In light of the high levels of lead in food in Guangzhou and the adverse effects of lead exposure on human health, it is essential to carry out a dietary exposure assessment for Guangzhou residents. However, health risks associated with such exposure in Guangzhou residents are currently not known. In general, dietary lead intake is related to age, with higher exposure levels in children than in other age groups since children consume more food per unit of body weight than the general adult population [37,38]. In view of the special food consumption patterns and the unique vulnerabilities of children, lead exposure in children is of particular concern. The objectives of this study were: (1) to estimate the dietary intake of lead in relation to age in Guangzhou, (2) to assess the corresponding health risks to the population using the MOE method, and (3) to identify the major contributing food categories to total lead exposure.

## 2. Materials and Methods

### 2.1. Chemicals and Instruments

This study was approved by the Research Ethics Committee from the Guangzhou Center for Disease Control and Prevention, Guangdong, China. Informed consent was obtained from all participants. The ultrapure water (resistivity 18.2 MΩ·cm) used in sample and solution preparation was obtained using a Milli-Q water purification system (Millipore Synergy, Carrollton, GA, USA). The nitric acid and 30% hydrogen peroxide used in this study were of ultrapure grade. Lead contents were determined by an inductively coupled plasma mass spectrometer equipped with collision cells (Agilent 7700 Series, Tokyo, Japan) operating with high-purity argon (99.999%, Guangzhou Air Plant, Guangzhou, China). The sample introduction system was composed of a quartz cyclonic spray chamber and a micromist nebulizer connected by Tygon^®^ tubes to the peristaltic pump of the inductively coupled plasma-mass spectrometry (ICP-MS, Agilent, Tokyo, Japan).

### 2.2. Food Sampling

Data on lead concentrations were derived from chemical pollutant surveillance data from the food safety risk monitoring system in Guangzhou that were obtained from 2014 to 2017. Samples were collected using a multistage-stratified sampling method from 12 districts of Guangzhou. Further details on data collection are available in the National Food Safety Risk monitoring manual [39]. Three streets were randomly selected in each district as monitoring sites. At each monitoring site, investigators, acting as consumers, purchased food samples from retailers including supermarkets, restaurants, agricultural product wholesale markets, and stores. A total of 6339 food samples were collected. The main food categories were rice and its products, wheat flour and its products, beans and its products, meat and its products, milk and dairy products, eggs and their products, vegetables, fruits, aquatic products, edible fungi, and algae. Samples were placed in sealed plastic bags and sent to the laboratory as soon as possible for cryopreservation until digestion.

### 2.3. Analytical Procedure

Food samples (0.3–0.8 g, accurate to 0.001 g) were weighed and placed in a PFA digestion vessel, and 5 mL of nitric acid and 2 mL of 30% hydrogen peroxide were then added. The vessel was placed inside a microwave decomposition system (MILESTONE ETHOS ONE, Bergamo, Italy), and decomposition was carried out according to the following program: (1) 120 °C (power 1000 W) for 5 min, (2) 150 °C (1000 W) for 10 min, (3) 190 °C (1000 W) for 20 min, and (4) 0 °C (0 W) for 20 min. The solutions were then left to cool down, and the volume was expanded to 25 mL with ultrapure water. At the same time, blank and reference tests were prepared. The ICP-MS settings and other operating conditions for the analysis are shown in Table 1.

### 2.4. Quality Assurance

Lead reference materials were certified as heavy metals and granted certificates by the Chinese scientific community. All vessels were soaked in a nitric solution (nitric acid: ultrapure water = 1:9) for 24 h and then rinsed with ultrapure water. National first-level standard material (GBW10035; Pb, Cd, and Cr in wheat powder, Chinese Academy of Geographical Sciences, Beijing, China; 1.63 ± 0.03 mg/kg for Pb) was used to determine quality assurance during the lead detection process. The results had a deviation of <5% from the GBW10035 wheat powder certified values. The limit of detection (LOD) for lead was 0.003 mg/kg, and the limit of quantification (LOQ) was 0.01 mg/kg. The percentage recovery of lead ranged from 85.2% to 108.7%.

### 2.5. Food Consumption Data

Food consumption data were sourced from a 2011 dietary survey of Guangzhou residents [40]. Dietary consumption information and general demographic information, including gender, age, occupation, and other characteristics, were collected for each respondent. A total of 2960 residents from 998 households were surveyed and 24-h dietary recalls were conducted on 3 consecutive days (two weekdays and one weekend day, holidays excluded). For those younger than 7 or older than 75 years of age, dietary information was obtained from adult family members. Among the respondents, 1416 were male (47.8%) and 1544 were female (52.2%); 1739 were from urban areas (58.8%) and 1221 were from suburban areas (41.2%). The sample included 253 preschool children aged 3–6 years (8.6%), 583 school children aged 7–17 years (19.7%), 1966 adults aged 18–59 years (66.4%), and 158 adults aged ≥60 years (5.3%).

### 2.6. Intake Calculation

Dietary lead intake was assessed using the point estimate method, as recommended by the FAO/WHO [41]. Total dietary lead intake was calculated by combining the fixed food consumption (such as the mean consumption or high consumption) with the fixed lead concentration in each food (here referred to as the mean concentration), and then summing the respective intakes from each food group. The following formula was used:

(1) $$Exp\sum_{k=1}^{n}\frac{Xk*Ck}{W}$$

where *Exp* (μg/kg bw/day) is the daily dietary lead intake of the studied population; *Xk* is the daily consumption of food *k*; *Ck* is the mean concentration of lead in food *k*, with non-detected results assigned to half the LOD, as recommended by the WHO [42]; *W* is the average body weight of the studied population; and *n* is the total number of food groups consumed.

The mean lead exposure and P95 (95th percentile) exposure were calculated for the overall study population and subgroups. The contribution of each food group to the total mean dietary lead intake was calculated for each age group.

### 2.7. Risk Assessment

As described above, there is no health-based guidance value for lead. Therefore, the MOE approach was used to assess the health risks of dietary lead exposure. The MOE is defined as the ratios of the observable effect level (e.g., the no observed adverse effect level (NOAEL) or benchmark dose lower bound (BMDL)) on the dose–response curve to the critical effect and the exposure level of the population. The European Food Safety Authority (EFSA) recommended using the benchmark dose (BMD) to obtain the MOE [31,43], i.e., MOE = BMDL/EXP. An MOE of less than 1 indicates a high health risk, whereas a MOE of greater than 1 indicates an acceptably low risk [44,45].

Both the JECFA and EFSA identified developmental neurotoxicity (IQ decrease) in young children and cardiovascular effects (SBP increase) in adults as the critical adverse effects of lead that could be used for risk assessment. In 2010, the JECFA concluded that a dietary exposure level of 1.2 μg/kg bw/day is associated with a population increase in SBP of 1 mmHg in adults, whereas dietary exposure of 0.6 μg/kg bw/day is associated with a population decrease of 1 IQ point in children. These dose estimates are not health-based guidance values, but rather levels below which the health risk is considered to be acceptably low.

### 2.8. Statistical Analysis

Microsoft Excel 2007 and IBM SPSS 20.0 (IBM Corp, Armonk, NY, USA) were used for the data summary and statistical analysis.

## 3. Results

### 3.1. Contaminant Monitoring and Food Consumption Data

Lead concentrations were below the LOD in 28.4% of the 6339 food samples, yielding a detection rate of 71.6%. Among the 27 food groups, the foods with the highest detectable rates of lead were meat products (100%), root and tuber vegetables (100%), dried seafood (100%), edible fungi (100%), and mollusks (99.7%). Milk and milk powder had low detection rates (3.4% and 22.8%, respectively), and detection rates in the remaining foods ranged from 57.6% to 89.3%. The mean concentration of lead in the 6339 samples was 0.0443 mg/kg, and the median and P95 values were 0.0130 and 0.1710 mg/kg, respectively. The food group with the highest average concentration of lead was algae (dry) (0.3947 mg/kg), followed by dried seafood (0.3219 mg/kg), mollusks (0.1384 mg/kg), edible fungi (0.0729 mg/kg), wheat flour and its products (0.0509 mg/kg), bulb vegetables (0.0505 mg/kg), meat products (0.0394 mg/kg), leafy vegetables (0.0354 mg/kg), rice and its products (0.0341 mg/kg), and preserved eggs (0.0341 mg/kg). Lead concentrations in all other food categories were lower than 0.02 mg/kg, with the lowest concentrations found in milk (0.0036 mg/kg) and milk powder (0.0071 mg/kg) (Table 2).

**Figure 1:**
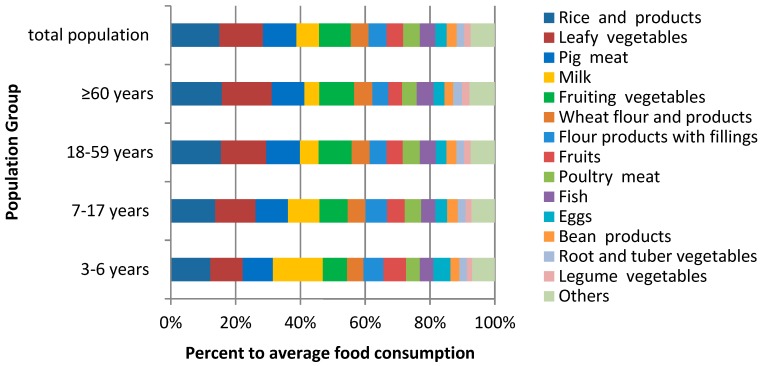
Average food consumption for various age groups.

Among preschool children, the food with the highest average consumption was milk, followed by rice and its products, wheat flour and its products, leafy vegetables, pork, and fruiting vegetables. In all other age groups, the food group with the highest average consumption was rice and its products, followed by leafy vegetables, wheat flour and its products (including products with fillings), pork, and fruiting vegetables (Figure 1). The portion of the population in the 95th percentile of food consumption had more than twice the average consumption, and this phenomenon was more evident for foods with lower overall consumption levels, but for some foods with extremely low consumption, the consumption in the 95th percentile was zero (Appendix A).

### 3.2. Dietary Lead Exposure and Risk Assessment

The mean dietary lead exposure in the overall population was 0.4033 μg/kg bw/day, with mean exposure values of 0.7466, 0.4739, 0.3759, and 0.4031 μg/kg bw/day in those aged 3–6, 7–17, 18–59, and ≥60 years, respectively. The 95th percentile of dietary lead exposure was 1.3049 μg/kg bw/day in the overall population and 2.4525, 1.5522, 1.1832, and 1.3589 μg/kg bw/day in the four age groups, respectively. As can be seen from the results, dietary lead exposure varied by age, with the highest exposure levels seen in young children. Lead intake decreased with increasing age, but a rising trend was observed for intake in the elderly (Table 3).

On the basis of BMDLs of 1.2 μg/kg bw/day for adults and 0.6 μg/kg bw/day for children, MOE values were calculated for each age group at the mean and 95th percentile exposure levels. These MOEs were 0.8 and 0.2 for preschool children, 1.3 and 0.4 for school children, 3.2 and 1.0 for adults, and 3.0 and 0.9 for the seniors, respectively. At mean exposure levels, the MOE values for all age groups were greater than 1 and less than 4, except in preschool children, for whom the MOE values were less than 1. The MOE value at the 95th percentile exposure level was less than or equal to 1 in all age groups. These findings indicate a low health risk of dietary lead exposure for Guangzhou residents as a whole, but high risk for young children and consumers of foods with high lead concentrations (Table 4).

### 3.3. Contributions of Different Food Groups to the Mean Lead Exposure

**Figure 2:**
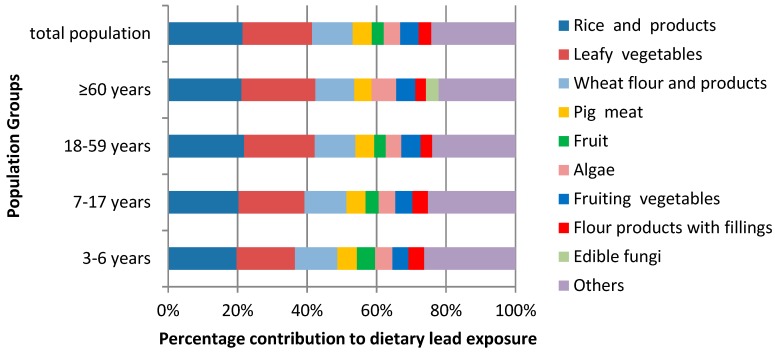
Population age groups and major food contributors to dietary lead exposure.

The contribution of each food category to the total dietary lead intake is shown in Table 3. In Figure 2, all food groups contributing less than 3% of lead exposure are merged into one group named “others”. The largest food sources of lead intake were found to be rice and rice products, leafy vegetables, and wheat flour and products, which together contributed 53.18% of the total lead intake in the overall population (48.73% for preschool children, 51.36% for school children, 53.90% for adults, and 53.60% for seniors). In addition to these three food groups, pork, fruit, algae, fruiting vegetables, and other flour products were also found to be significant dietary sources of lead exposure, with contributions varying by population age group. It is worth noting that young children and the elderly, both vulnerable groups, showed significant differences from the other age groups in terms of the contribution rate of several foods to dietary lead exposure. Although the contribution of leafy vegetables in preschool children was 16.78%, it was still lower than in the other age groups. Conversely, the contribution of fruits in preschool children (5.29%) was higher than that in all other age groups (≤3.78%). The most striking difference between the elderly and other age groups was the relatively high contribution of algae and edible fungi to dietary lead exposure in those aged ≥60 years. Algae contributed 7.07% to dietary lead exposure in this age group, compared to less than 5% in all other age groups.

## 4. Discussion

Among all food categories, the highest lead concentrations in Guangzhou were found in algae (dry), followed by dried seafood and mollusks. These patterns were different from those observed in Guangdong province [46] and Shenzhen city [47], where preserved eggs had the highest lead levels. One possible reason for the relatively low lead concentration in preserved eggs in our study was a reform of the production process for preserved eggs involving the replacement of lead oxide with zinc oxide in the process of soaking eggs with feed liquid. The mean lead concentration in rice and its products, the staple food and main energy source for the vast majority of residents in Guangzhou, was 0.0341 mg/kg. This was lower than the levels reported in Guangdong province (0.0495 mg/kg) [46] and Shanghai (0.071 mg/kg) [48], but higher than those reported in Shenzhen (0.018 mg/kg) [49].

Dietary lead intake is dependent on both the concentration of lead in food and the amount of food consumed. In this study, lead intake was calculated using available food consumption data and food lead concentration data. As children form a special population whose food consumption patterns differ from those in other age groups, we carried out separate exposure assessments by age group. Studies similar to ours have been conducted in other parts of China. Sun et al. [50] estimated the dietary intake of lead in Jiangsu Province using the diary study method and found a mean intake of 2.75 μg/kg bw/day for children aged 2–6 years and 1.55 μg/kg bw/day for the general population. Jin et al. [44] re-estimated the dietary lead exposure in Jiangsu using new contaminant surveillance data, and their results indicated a dietary lead intake of 3.019 μg/kg bw/day in children aged 2–6 years and 1.742 μg/kg bw/day in the general population. Fan et al. [51] reported dietary lead exposure values for children aged 2–6 years and for the general population in Shaoxing of 1.87 and 1.14 μg/kg bw/day, respectively. In our study, the mean dietary lead exposure was 0.7466 for children aged 3–6 years and 0.4033 μg/kg bw/day for the general population. These values are substantially lower than those reported in the other studies described above.

Generally, a total diet study (TDS) using mean food consumption and mean contaminant concentration provides a point estimate of dietary exposure that is comparable to the mean estimate from a food diary study [52]. Therefore, in addition to comparing our results with those obtained elsewhere using the same research method, we compared the mean values with the results from the TDS (Table 5). The dietary lead intake in Guangzhou city was lower than that in Shenzhen city [47], which is near Guangzhou and has a similar geographical environment. According to 2007 TDS data from China, the dietary intake of lead was 2.54 μg/kg bw/day for children 2–7 years old, 1.73 μg/kg bw/day for adults aged 20–50 years old, and 1.52 μg/kg bw/day for adults aged over 65 [34]. These values are higher than the dietary lead intake values found in our study. This discrepancy may be due to measures taken by the Chinese government since 2007 to reduce environmental lead pollution. Hong Kong, a coastal city in the southeast of China, is also adjacent to Guangzhou. In 2014, Chen et al. [53] estimated the dietary lead intake among adults in this region using the TDS method and reported a mean intake of 0.21 μg/kg bw/day, which is substantially lower than that found in our study. The dietary lead intake in Guangzhou was close to or higher than that found in several other countries [33,38,45,54,55,56], but was lower than that observed in Eastern Poland [57], Serbia [58], and Italy [59]. It is important to note, however, that such comparisons should be interpreted cautiously because of differences in survey timing, food consumption patterns, research methodology, the age range of the studied population, the food categories included, the methods used to collect consumption data and to analyze contaminants, and other differences between studies.

Our study isolated the elderly from the entire population for a separate dietary exposure assessment. This method was relatively novel, as most other studies have classified the elderly with other adults. The mean intake of lead in the elderly (0.4031 μg/kg bw/day) was slightly higher than that in adults (0.3759 μg/kg bw/day). Possible reasons for this discrepancy include: (1) the average food consumption on the basis of per unit of body weight of the seniors being greater than that of adults; and (2) that the elderly were consuming more food containing a high concentration of lead, such as algae and edible fungi. In our study, the MOE values for the average dietary lead exposure were 0.8 for 3–6 years, 1.3 for 7–17 years, 3.2 for 18–59 years, and 3.0 for ≥60 years. A dietary lead exposure assessment in the Chinese population conducted by Li et al. yielded MOE values of 0.1 for those aged 2–12 years, 0.7 for those aged 13–19 years, and 0.7–0.9 for those at least 20 years of age [34]. For the residents of Jiangsu province, the MOE values for the mean lead exposure were found to be 0.272 for those aged 2–6 years and 1.031 for those aged 18–80 years [44]. The MOE values of dietary lead exposure in Shaoxing were found to be 0.32–0.35 for those aged 2–6 years, 0.49–0.59 for those aged 7–17 years, and 1.3–1.7 for those aged ≥18 years [51]. The MOE values at the mean lead exposure level in the Australian population were found to be 1.1 for those aged 2–5 years, 1.7 for those aged 6–12 years, 10 for those aged 13–16 years, and 9.2 for those aged ≥17 years [33]. It can be seen from the above results that the MOE values in Guangzhou reported in the present study are larger than those reported for Jiangsu, Shaoxing, and China overall, but smaller than those reported for Australia. A higher MOE value indicates a lower health risk from exposure; thus, the health risk of dietary lead exposure for Guangzhou residents appears to be lower than that in Jiangsu, Shaoxing, and China overall, but higher than that in Australia. Based on the evaluation criteria of MOE, children and high food consumers in Guangzhou have a high health risk, while others have a low risk.

The principal food sources of lead for Guangzhou residents were found to be rice and its products, leafy vegetables, wheat flour and its products, pork, fruiting vegetables, algae, and fruits. This pattern is different from that observed in Shenzhen [47], where the major food sources of lead were found to be eggs and egg products, fish and seafood and related products, vegetables and vegetable products, and meat and meat products. The amount of algae consumed was very small in most areas, and algae were therefore not included in these studies. However, due to the unique geographical location of Guangzhou, seafood such as algae is easily available to residents and is a popular food. Accordingly, algae were consumed far more frequently in Guangzhou than in other areas in China such as Shaoxing [51]. Our results indicate that lead intake from algae cannot be ignored in the Guangzhou region, especially among elderly residents, for whom the contribution rate of algae to the total dietary lead intake was found to be 7.07%.

## 5. Conclusions

This study is the first to systematically evaluate dietary lead exposure by age group in Guangzhou. Rice and rice products, leafy vegetables, and wheat flour and its products were found to be the largest food sources of lead intake, followed by pork, fruiting vegetables, algae, and fruit. The health risks of dietary lead exposure were measured using the MOE approach. The MOE was less than 1 for young children and for individuals with high consumption levels of certain food groups, while it was greater than 1 for the rest of the population. Accordingly, the health risk associated with dietary lead exposure is low for the majority of the population in Guangzhou, but there are high risks for young children and consumers of foods with high lead content. Efforts are still needed to reduce lead exposure. This study provides a preliminarily assessment of dietary lead exposure in Guangzhou using the point evaluation method. However, because of differences in food consumption and body weight between individuals, a more accurate probabilistic approach is needed to further characterize dietary lead exposure in this population. Furthermore, the use of food consumption data collected over a three-day period may not accurately reflect the dietary habits of residents, and dietary data collected over a longer time period and across multiple seasons would provide a more accurate measure of dietary intake and corresponding lead exposure. Finally, the dietary survey was performed before the assessment of lead content in food, and food consumption in 2011 may not be exactly equal to consumption in 2014–2017, which may have added bias to the results. However, dietary intake patterns tend to be relatively stable over time, and dietary intake data from 2011 therefore likely provided an accurate proxy for food consumption in 2014–2017.

## Figures and Tables

**Table 1:** Summary of inductively coupled plasma-mass spectrometry (ICP-MS) operating conditions.

Parameters	Operating Conditions
RF Power (KW)	1550
Sample Depth (mm)	8.0
Carrier Gas (L/min)	0.7
Makeup Gas (L/min)	0.5
Argon Flow (mL/min)	4.3
Extraction 1 (V)	0.0
Extraction 2 (V)	−130
Omega Bias (V)	−90
Omega Lens (V)	7.0
Deflect (V)	1.4
Octopole Bias (V)	−18
Octopole RF (V)	140
KED (V)	3.0

**Table 2:** Summary of lead concentrations in food, Guangzhou City, 2014–2017 (mg/kg).

Food Category	Samples	Mean	SD ^a^	P50	P95	>LOD (%)
Rice and rice products	990	0.0341	0.0430	0.0151	0.1341	73.2
Wheat flour and products	1165	0.0509	0.0634	0.0260	0.1707	81.7
Flour products with fillings	26	0.0158	0.0118	0.0130	0.0427	88.5
Other cereals	479	0.0206	0.0264	0.0090	0.0820	57.6
Pulses	110	0.0272	0.0262	0.0200	0.0700	80.0
Bean products	110	0.0151	0.0245	0.0089	0.0645	74.5
Preserved eggs	34	0.0341	0.1646	0.0040	0.2660	64.7
Eggs	306	0.0099	0.0115	0.0060	0.0307	68.6
Pig meat	221	0.0127	0.0122	0.0100	0.0300	69.7
Livestock meat ^b^	86	0.0083	0.0113	0.0045	0.0356	62.8
Poultry	99	0.0115	0.0162	0.0090	0.0280	83.8
Edible offal	140	0.0240	0.0319	0.0190	0.0700	89.3
Meat products	72	0.0394	0.0262	0.0345	0.0837	100.0
Milk powder	145	0.0071	0.0081	0.0050	0.0200	22.8
Milk	208	0.0036	0.0023	0.0500	0.0050	3.4
Leafy vegetables ^c^	356	0.0354	0.0464	0.0170	0.1432	77.2
Fruiting vegetables ^d^	163	0.0130	0.0167	0.0060	0.0468	62.0
Root and tuber vegetables	5	0.0230	0.0124	0.0220	0.3500	100.0
Legume vegetables	115	0.0209	0.0360	0.0050	0.1096	62.6
Bulb vegetables	42	0.0505	0.0616	0.0270	0.2140	64.3
Fruit	223	0.0160	0.0181	0.0090	0.0594	67.3
Fish	536	0.0134	0.0178	0.0068	0.0483	70.9
Crustaceans	143	0.0109	0.0101	0.0074	0.0322	68.5
Mollusks	318	0.1384	0.1117	0.1200	0.3351	99.7
Dried seafood	24	0.3219	0.2603	0.2365	0.9000	100.0
Algae	175	0.3947	0.4349	0.3400	0.8938	78.9
Edible fungi	48	0.0729	0.0147	0.0750	0.0900	100.0
Total	6339	0.0433	0.1104	0.0130	0.1710	71.6

Notes: ^a^ Standard deviation; ^b^ livestock meat excluding pork; ^c^ leafy vegetables including immature stem vegetables and Brassica vegetables; ^d^ fruiting vegetables include eggplant, tomato, green pepper, cucumber, cucurbit, and others. LOD: limit of detection; P50: 50th percentile; P95: 95th percentile.

**Table 3:** Detailed estimated dietary exposure (mean and 95th percentile) to lead among Guangzhou residents (μg/kg bw/day).

Food Category	3–6 Years			7–17 Years			18–59 Years			≥60 Years			Total Population		
	Mean	P95	Contrib.	Mean	P95	Contrib.	Mean	P95	Contrib.	Mean	P95	Contrib.	Mean	P95	Contrib.
Rice and rice products	0.1473	0.3163	19.73%	0.0960	0.2104	20.26%	0.0822	0.1733	21.87%	0.0851	0.1677	21.11%	0.0864	0.1865	21.42%
Wheat flour and products	0.0912	0.3057	12.22%	0.0572	0.1969	12.07%	0.0438	0.1396	11.65%	0.0450	0.1371	11.16%	0.0474	0.1519	11.75%
Flour products with fillings	0.0343	0.1090	4.59%	0.0214	0.0682	4.52%	0.0127	0.0414	3.38%	0.0123	0.0452	3.05%	0.0147	0.0476	3.64%
Other cereals	0.0071	0.0342	0.95%	0.0033	0.0183	0.70%	0.0031	0.0156	0.82%	0.0051	0.0231	1.27%	0.0034	0.0173	0.84%
Pulses	0.0027	0.0232	0.36%	0.0026	0.0169	0.55%	0.0017	0.0108	0.45%	0.0010	0.0076	0.25%	0.0018	0.0118	0.45%
Bean products	0.0147	0.0691	1.97%	0.0101	0.0514	2.13%	0.0072	0.0331	1.92%	0.0064	0.0313	1.59%	0.0078	0.0376	1.93%
Preserved eggs	0.0009	0	0.12%	0.0006	0.0026	0.13%	0.0004	0.0019	0.11%	0.0003	0.0001	0.07%	0.0004	0.0021	0.10%
Eggs	0.0183	0.0548	2.45%	0.0075	0.0187	1.58%	0.0051	0.0134	1.36%	0.0052	0.0132	1.29%	0.0059	0.0154	1.46%
Pig meat	0.0419	0.1046	5.61%	0.0260	0.0624	5.49%	0.0206	0.0490	5.48%	0.0202	0.0485	5.01%	0.0220	0.0540	5.45%
Livestock meat	0.0035	0.0161	0.47%	0.0026	0.0127	0.55%	0.0022	0.0091	0.59%	0.0014	0.0070	0.35%	0.0022	0.0103	0.55%
Poultry	0.0174	0.0511	2.33%	0.0119	0.0304	2.51%	0.0094	0.0263	2.50%	0.0082	0.0213	2.03%	0.0100	0.0286	2.48%
Edible offal	0.0036	0.0233	0.48%	0.0027	0.0185	0.57%	0.0023	0.0131	0.61%	0.0025	0.0202	0.62%	0.0024	0.0149	0.60%
Meat products	0.0115	0.0721	1.54%	0.0092	0.0526	1.94%	0.0057	0.0324	1.52%	0.0044	0.0266	1.09%	0.0063	0.0368	1.56%
Milk powder	0.0025	0.0203	0.33%	0.0001	0	0.02%	0	0	0.00%	0.0001	0	0.02%	0.0001	0	0.02%
Milk	0.0195	0.0613	2.61%	0.0072	0.0213	1.52%	0.0032	0.0130	0.85%	0.0026	0.0105	0.65%	0.0043	0.0168	1.07%
Leafy vegetables	0.1253	0.3242	16.78%	0.0902	0.2053	19.03%	0.0766	0.1751	20.38%	0.0860	0.2082	21.33%	0.0807	0.1968	20.01%
Fruiting vegetables	0.0344	0.0912	4.61%	0.0231	0.0604	4.87%	0.0207	0.0533	5.51%	0.0222	0.0622	5.51%	0.0215	0.0574	5.33%
Root and tuber vegetables	0.0191	0.0766	2.56%	0.0113	0.0445	2.38%	0.0084	0.0359	2.23%	0.0100	0.0407	2.48%	0.0092	0.0393	2.28%
Legume vegetables	0.0117	0.0574	1.57%	0.0079	0.0323	1.67%	0.0065	0.0285	1.73%	0.0077	0.0293	1.91%	0.0070	0.0296	1.74%
Bulb vegetables	0.0086	0.0758	1.15%	0.0053	0.0383	1.12%	0.0047	0.0277	1.25%	0.0059	0.0310	1.46%	0.0049	0.0314	1.21%
Fruit	0.0395	0.1403	5.29%	0.0179	0.0708	3.78%	0.0124	0.0512	3.30%	0.0108	0.0411	2.68%	0.0140	0.0561	3.47%
Fish	0.0199	0.0613	2.67%	0.0119	0.0316	2.51%	0.0102	0.0294	2.71%	0.0111	0.0275	2.75%	0.0108	0.0303	2.68%
Crustaceans	0.0018	0.0160	0.24%	0.0012	0.0084	0.25%	0.0011	0.0072	0.29%	0.0012	0.0080	0.30%	0.0012	0.0074	0.30%
Mollusks	0.0061	0.0509	0.82%	0.0061	0.0536	1.29%	0.0050	0.0385	1.33%	0.0051	0.0605	1.27%	0.0052	0.0431	1.29%
Dried seafood	0.0053	0	0.71%	0.0045	0	0.95%	0.0032	0	0.85%	0	0	0.00%	0.0030	0	0.74%
Algae	0.0369	0.1908	4.94%	0.0229	0.1648	4.83%	0.0169	0.1084	4.50%	0.0285	0.2209	7.07%	0.0191	0.1230	4.74%
Edible fungi	0.0216	0.1069	2.89%	0.0132	0.0609	2.79%	0.0106	0.0560	2.82%	0.0148	0.0701	3.67%	0.0116	0.0589	2.88%
Total	0.7466	2.4525	100.00%	0.4739	1.5522	100.00%	0.3759	1.1832	100.00%	0.4031	1.3589	100.00%	0.4033	1.3049	100.00%

**Table 4:** Summary of margin of exposure (MOE) by age group in Guangzhou city.

Age Groups	MOE (Mean Exposure)	MOE (P95 Exposure)
3–6 years ^a^	0.8	0.2
7–17 years ^a^	1.3	0.4
18–59 years ^b^	3.2	1.0
≥60 years ^b^	3.0	0.9

^a^ Results derived from the benchmark dose lower bound (BMDL) equal to 0.6 μg/kg bw/day. ^b^ Results derived from the BMDL equal to 1.2 μg/kg bw/day.

**Table 5:** Comparison of dietary lead exposure in Guangzhou with other data from the total diet study.

Country	Population Group	Mean Exposure (μg/kg bw/day)	Reference
China (Guangzhou)	3–6 years	0.7466	Present study
	7–17 years	0.4739	
	18–59 years	0.3759	
	≥60 years	0.4031	
	Overall population	0.4033	
Canada (Ontario)	Adult	0.21	Juric et al. (2018)
China (Shenzhen)	Adult	0.59–0.73 ^a^	Pan et al. (2016)
Eastern Poland	Young adult	0.79	Koch et al. (2016)
China (Hong Kong)	Adult	0.21	Chen et al. (2014)
Serbia	Adult	1.03	Skrbic et al. (2013)
Korea	Overall population	0.183	Koh et al. (2012)
China	2–7 years	2.54	Li et al. (2012)
	8–12 years	2.50	
	13–19 years	1.75	
	20–50 years	1.73	
	51–65 years	1.54	
	>65 years	1.52	
European countries	Children	0.96 ^b^	EFSA (2012)
	Adolescent	0.55	
	Adult	0.50	
	Elderly	0.49	
	Overall population	0.68	
France	Children	0.27	Arnich et al. (2012)
	Adult	0.2	
Australian	2–5 years	0.27 ^c^	FSANZ (2011)
	6–12 years	0.18	
	13–16 years	0.12	
	≥17 years	0.13	
United Kingdom	Adult	0.09–0.10 ^a^	Rose et al. (2010)
Lebanon	Adult	0.11	Nasreddine et al. (2010)
Italy (Pavia)	Adult	0.85	Turconi et al. (2009)

^a^ Exposure data of lower and upper bound estimates are presented as a range based on assigning zero and the LOD, respectively, to instances of non-detection. ^b^ Dietary exposure based on the middle bound mean lead occurrence. ^c^ Using the median concentration of Pb and assigning 0 to instances of non-detection. EFSA: European Food Safety Authority.

## Appendix A

**Table A1:** Food consumption among Guangzhou residents in 2011 (g/day).

Food Category	3–6 years		7–17 years		18–59 years		≥60 years		Overall Population	
	Mean	P95	Mean	P95	Mean	P95	Mean	P95	Mean	P95
Rice and rice products	78.6 ± 45.5	168.8	121.3 ± 73.9	265.9	146.5 ± 90.8	309.0	148.5 ± 89.6	292.6	135.8 ± 86.9	293.2
Wheat flour and products	32.6 ± 42.3	109.3	48.4 ± 58.6	166.7	52.3 ± 58.8	166.7	52.6 ± 62.0	160.3	49.9 ± 57.9	160.0
Flour products with fillings	39.5 ± 42.8	125.5	58.5 ± 64.5	186.0	48.9 ± 58.3	159.4	46.2 ± 53.5	170.1	49.9 ± 58.4	161.6
Other cereals	6.3 ± 18.8	30.2	7.0 ± 17.5	38.3	9.2 ± 27.5	46.0	14.8 ± 42.3	66.7	8.8 ± 26.3	45.0
Pulses	1.8 ± 5.4	15.5	4.1 ± 15.6	26.7	3.8 ± 14.0	24.2	2.2 ± 9.0	16.7	3.6 ± 13.6	23.3
Bean products	17.7 ± 32.7	83.3	28.8 ± 49.6	146.7	28.9 ± 50.0	133.3	25.1 ± 41.6	123.3	27.7 ± 48.3	133.3
Preserved eggs	0.5 ± 3.3	0.0	0.7 ± 4.5	3.3	0.7 ± 3.4	3.3	0.5 ± 3.0	0.2	0.6 ± 3.6	3.3
Eggs	33.7 ± 30.1	100.8	32.5 ± 30.1	81.2	31.3 ± 29.6	82.6	31.2 ± 25.9	79.5	31.7 ± 29.6	83.3
Pig meat	60.0 ± 43.2	149.9	88.4 ± 64.4	211.6	98.6 ± 69.9	234.4	94.5 ± 62.9	227.0	93.0 ± 67.4	228.0
Livestock meat	7.6 ± 15.4	35.3	13.6 ± 23.0	66.0	16.0 ± 26.7	66.7	9.9 ± 17.3	50.0	14.5 ± 24.9	66.7
Poultry	27.6 ± 31.6	80.9	44.7 ± 40.6	114.1	49.9 ± 48.0	139.1	42.3 ± 36.8	110.4	46.6 ± 45.3	133.3
Edible offal	2.7 ± 9.9	17.7	4.8 ± 13.8	33.3	5.7 ± 16.7	33.3	6.3 ± 17.1	50.0	5.3 ± 15.7	33.3
Meat products	5.3 ± 12.4	33.3	10.1 ± 20.3	57.5	8.8 ± 18.8	50.0	6.6 ± 15.7	40.2	8.6 ± 18.5	50.0
Milk powder	6.4 ± 17.1	52.0	0.5 ± 4.8	0.0	0.2 ± 2.0	0.0	0.6 ± 4.8	0.0	0.8 ± 6.0	0.0
Milk	98.7 ± 111.4	309.7	85.7 ± 102.0	255.3	53.8 ± 79.0	218.8	43.1 ± 64.4	173.6	63.4 ± 88.1	250.0
Leafy vegetables	64.4 ± 54.4	166.7	109.8 ± 75.4	250.0	131.5 ± 93.2	300.8	144.6 ± 98.4	350.0	122.2 ± 89.7	298.0
Fruiting vegetables	48.1 ± 41.4	127.7	76.6 ± 65.1	200.3	96.6 ± 83.7	249.5	101.5 ± 101.9	284.8	88.8 ± 80.0	236.7
Root and tuber vegetables	15.1 ± 25.2	60.6	21.2 ± 34.9	83.3	22.1 ± 36.5	94.9	25.9 ± 36.5	105.3	21.5 ± 35.4	91.6
Legume vegetables	10.2 ± 17.9	50.0	16.2 ± 25.9	66.7	19.0 ± 30.2	82.9	22.0 ± 39.3	83.5	17.9 ± 29.2	76.0
Bulb vegetables	3.1 ± 9.5	27.3	4.5 ± 12.6	32.7	5.6 ± 14.0	33.3	6.9 ± 13.9	36.5	5.2 ± 13.4	33.3
Fruit	44.9 ± 58.8	159.6	48.3 ± 71.6	190.8	47.2 ± 69.3	194.4	40.1 ± 58.4	152.8	46.9 ± 68.4	187.9
Fish	27.0 ± 29.7	83.3	38.3 ± 35.9	101.6	46.1 ± 42.9	133.3	49.3 ± 46.4	122.1	43.1 ± 41.2	121.0
Crustaceans	3.0 ± 8.6	26.7	4.9 ± 13.6	33.3	6.3 ± 20.4	40.0	6.7 ± 17.5	43.7	5.7 ± 18.4	36.6
Mollusks	0.8 ± 4.2	6.7	1.9 ± 7.8	16.7	2.2 ± 8.9	16.9	2.2 ± 7.9	26.0	2.0 ± 8.4	16.7
Dried seafood	0.3 ± 1.8	0.0	0.6 ± 4.6	0.0	0.6 ± 4.6	0.0	0.0 ± 0.3	0.0	0.5 ± 4.3	0.0
Algae	1.7 ± 7.4	8.8	2.5 ± 8.3	18.0	2.6 ± 9.6	16.7	4.3 ± 15.4	33.3	2.6 ± 9.6	16.7
Edible fungi	5.4 ± 9.8	26.7	7.8 ± 17.5	36.0	8.8 ± 18.8	46.7	12.1 ± 20.4	57.2	8.5 ± 18.1	43.3
